# Conferring Antioxidant Activity to an Antibacterial and Bioactive Titanium Surface through the Grafting of a Natural Extract

**DOI:** 10.3390/nano13030479

**Published:** 2023-01-25

**Authors:** Francesca Gamna, Seiji Yamaguchi, Andrea Cochis, Sara Ferraris, Ajay. Kumar, Lia Rimondini, Silvia Spriano

**Affiliations:** 1 DISAT Department, Politecnico di Torino, Corso Duca degli Abruzzi 24, 10129 Torino, Italy; 2 Department of Biomedical Sciences, College of Life and Health Sciences, Chubu University, 1200 Matsumoto, Kasugai 487-8501, Aichi, Japan; 3 Department of Health Sciences, Center for Translational Research on Autoimmune and Allergic Diseases—CAAD, Università Del Piemonte Orientale UPO, Corso Trieste 15/A, 28100 Novara, Italy

**Keywords:** titanium, iodine, tea polyphenols, antibacterial, bioactive, anti-inflammatory

## Abstract

The main unmet medical need of bone implants is multifunctional activity, including their ability to induce rapid and physiological osseointegration, counteract bacterial biofilm formation, and prevent in situ chronic inflammation at the same time. This research starts from an already developed c.p. titanium surface with proven bioactive (in vitro hydroxyl apatite precipitation) and antibacterial activities, due to a calcium titanate layer with nano- and micro-scale roughness and loaded with iodine ions. Here, antioxidant ability was added to prevent chronic inflammation by grafting polyphenols of a green tea extract onto the surface, without compromising the other functionalities of the surface. The surface was characterized before and after functionalization through XPS analysis, zeta potential titrations, ion release measurements, in vitro bioactivity tests, SEM and fluorescence microscopy, and Folin–Ciocalteu and biological tests. The presence of grafted polyphenols as a homogeneous layer was proven. The grafted polyphenols maintained their antioxidant ability and were anchored to the surface through the linking action of Ca^2+^ ions added to the functionalizing solution. Iodine ion release, cytocompatibility towards human mesenchymal stem cells (hMSC), and antibacterial activity were maintained even after functionalization. The antioxidant ability of the functionalized surface was effective in preserving hMSC viability in a chemically induced pro-inflammatory environment, thus showing a scavenger activity towards toxic active species responsible for inflammation.

## 1. Introduction

Titanium and its alloys are widely used as materials for biomedical and prosthetic engineering, particularly for bone repair where large loads are involved, given their high mechanical properties. However, loosening of these implants is often reported, as bare, polished titanium has poor adhesion to bone and often requires a long period to achieve osseointegration [1]. Since the 1960s, when osseointegration was first observed, several surface modifications have been explored to give titanium and its alloys the ability to bond to bone following different treatments, such as chemical [2,3], mechanical [4], hydrothermal [5], ion implantation [6], and anodic oxidation [7].

Despite the positive results of increased bone bonding and bone growth achieved with the various surface treatments, the challenge of providing titanium with antibacterial activity remains an unmet medical issue. In fact, the incidence of prosthetic joint infection is still around 2% and it is continuously increasing [8]. To combat infections caused by pathogenic bacteria, antibiotic therapy is a widely used approach [9]. However, the main problem associated with antibiotic therapies is the increasing development of antibiotic-resistant bacterial strains and their narrow spectrum of activity, resulting in difficulty in treating infections caused by different bacteria [10]. For this reason, the scientific community began to investigate ways to make the titanium surface not only osseointegrated, but also antibacterial, through the use of different methods and antibacterial agents [11]. Current techniques to reduce bacterial attachment and biofilm formation include antiadhesive function and bactericidal function [12]. The incorporation of metal ions, particularly silver, copper, and zinc, which have antibacterial action, is of great interest [13,14,15,16,17]. Unfortunately, a proper balance between effective antibacterial action and cytocompatibility is difficult to be found [18]. Attention then shifted to iodine ions as excellent candidates for antibacterial, biocompatible, and noncytotoxic agents. When an iodine-containing calcium titanate is produced on Ti by a chemical and heat treatment, the treated metal exhibits in vitro and in vivo antibacterial activity, in vitro apatite formation, and in vivo bone bonding ability [19,20,21,22].

Another important factor that can lead to implant failure is inflammation; 47% of early implant failures are caused by inflammation [23]. Again, the scientific literature has focused on the search for surface functionalization or coatings on titanium that can modulate inflammation to achieve a multifunctional surface [24,25,26,27,28]. One area of research of significant interest is the functionalization of titanium surfaces for biomedical implants with polyphenols. Polyphenols are a diverse group of phytochemicals found in fruits, vegetables, nuts, and other plant-based foods. They have potent antioxidant properties and anti-inflammatory effects in the body [29].

One of the main mechanisms by which polyphenols exert their antioxidant effects is by redox chemical action and scavenging free radicals. Free radicals are highly reactive molecules that can cause damage to cells and contribute to the development of chronic diseases such as cancer and heart disease. Polyphenols act as antioxidants and neutralize these free radicals, thereby preventing cellular damage [30,31].

In addition to their antioxidant properties, polyphenols also have anti-inflammatory effects on the body. Inflammation is a natural response to injury or infection, but chronic inflammation is linked to the development of many diseases. Polyphenols inhibit the production of inflammatory molecules and modulate the activity of inflammatory cells, which can help to reduce inflammation in the body [32].

More controversial and still under study, is the antibacterial role of polyphenols. Studies can be found in the literature claiming the high antibacterial properties of polyphenols against certain bacteria strains [33,34,35]. Research on polyphenols also shows that they may have beneficial effects on a wide range of health conditions, including cancer, heart disease, diabetes, and neurodegenerative diseases [36,37,38,39].

Indeed, polyphenols have gained great attention in different industrial fields [40] due to their antioxidant, antibacterial, and anticarcinogenic capabilities. Their antioxidant action is strictly connected to the removal of radical oxygen species, and it resolves into an anti-inflammatory behavior in a biological environment. Polyphenols are molecules that can be extracted from a large number of natural products, including waste products, economically and sustainably, representing an important approach to the ecological use of resources. In this paper, polyphenols extracted from green tea are specifically studied. The polyphenol family includes a wide range of substances containing one or more phenolic groups. All polyphenols are classified into two main categories: phenolic acids and flavonoids [41]. Polyphenols contain both monomers and polymerized polyphenols. Green tea is very rich in catechins and their oligomers, which are part of the flavonoid family and are responsible for its renowned potential benefits, including antioxidant capacity. Among all catechins, epigallocatechin-3-gallate (EGCG) accounts for 60% of the catechins present in green tea [42].

In this work, a surface functionalization protocol was optimized to graft tea polyphenols onto a titanium surface previously made bioactive (in vitro precipitation of hydroxyapatite) through a specific heat treatment and made antibacterial through loading of iodine ions. These substrates have been extensively characterized in previous works and their osseointegration ability is already verified. In fact, a high long-term sustainable bone-bonding capacity was confirmed by in vivo tests and mechanical and histological experiments [19,22]. In addition, the same type of green tea used here (TPH) has been previously tested and was found to have good ability to promote osteoblast differentiation and mineralization [43].

For functionalization, a specific focus was paid to the selection of the solvent medium and pH, the effect of Ca^2+^ ions in the solution on the grafting mechanism, and the concentration of the polyphenol source solution. A preliminary characterization of the functionalized surfaces was performed using the Folin–Ciocalteu photometric test and XPS (X-ray photoelectron spectroscopy) analysis, to verify the presence and activity of the grafted polyphenols. In vitro bioactivity, antibacterial activity, cytocompatibility, and antioxidant capacity were evaluated in the functionalized samples. Accordingly, the formation of apatite was observed, the ability to prevent the surface colonization from the pathogen *Escherichia coli* was verified, and the antioxidant activity was confirmed by the possibility to protect human cells from toxic active species in a pro-inflammatory environment by a scavenger mechanism.

This research aims to improve an already extensively studied surface with exceptional osseointegration capacity and antimicrobial properties by incorporating the renowned anti-inflammatory properties of tea polyphenols to create a multifunctional surface with osseointegration and antimicrobial and anti-inflammatory capabilities.

## 2. Materials and Methods

### 2.1. Polyphenols Extraction

The polyphenols used in this work were extracted from green tea leaves (Longjing, produced in Hangzhou, China) before being used for functionalization. The extraction procedure, which had already been performed and described in previous work [28] involves the use of a solvent, ethanol, as the extraction medium. The extracted natural polyphenols were named TPH.

### 2.2. Surface Treatment

Commercially pure titanium (Ti) samples of rectangular shape (10 × 10 × 1 mm^3^) were polished with a 400 grain diamond plate. After being cleaned with acetone, 2-propanol, and ultrapure water in an ultrasonic bath for 30 min each, they were dried at 40 °C in an incubator overnight. Following this, a thermal–chemical surface treatment was performed, obtaining a calcium titanate surface layer. Briefly, the samples were initially immersed in an aqueous solution of NaOH (5 mol/L) at 60 °C for 24 h, and then in CaCl_2_ (100 mmol/L) at 40 °C for 24 h in a shaking oil bath at 120 rpm. The samples were then heated to 600 °C for 1 h. To introduce iodine ions on the surface, some samples were immersed in an iodine-containing solution of 10 mM/L ICl_3_ and then placed in an oil-shaking bath at 80 °C for 24 h. Other samples were placed in water at 80 °C for 24 h and used as controls. For bacterial tests, however, the treatments were conducted in the same way as described above, but the samples were larger (25 × 25 × 1 mm^3^).

The prepared samples were named Ti_Ca+I and Ti_Ca, respectively.

### 2.3. Surface Functionalization

**Figure 1:**
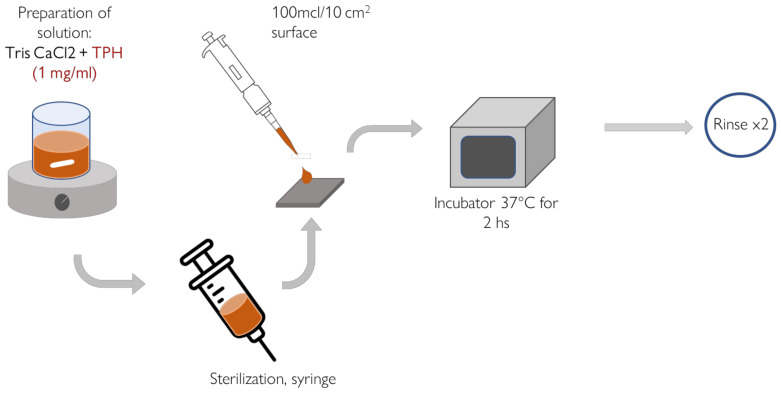
Method of functionalization with the TPH solution.

Ti_Ca and Ti_Ca+I samples were then functionalized in clean conditions with TPH, by placing a 100 µL/10 mm^2^ drop of TPH solution on the surface for 3 h at 37 °C in the dark. The TPH solution for functionalization was obtained with a concentration of 1 mg/mL, according to [44], by dissolving TPH in TRIS/HCl at 7.4 pH and stirring for 1 h. After the solution was prepared and the polyphenols were well dissolved, the solution was filtered with a syringe and a 0.2 µm filter to avoid bacterial contamination. The filtered solution was used for functionalization, which lasted 2 h. Finally, the samples were rinsed with double-distilled water twice and dried in a fumehood. The functionalized samples were named Ti_Ca+TPH and TI_Ca+I+TPH. The method of surface functionalization is illustrated in Figure 1.

### 2.4. X-ray Photoelectron Spectroscopy

The surface chemical composition and the surface of the functionalized and non-functionalized samples (Ti_Ca+I+TPH and Ti_Ca+I) were analyzed using X-ray photoelectron spectroscopy (XPS, PHI 5000 Versaprobe II, ULVAC-PHI, Inc., Kanagawa, Japan) with an Al-Kα beamline as the X-ray source at a take-off angle of 45°. The binding energy of the observed spectra was calibrated by referring to the C1s peak of the CH2 groups on the substrate, which occurs at 284.8 eV.

### 2.5. Zeta Potential Measurements

The zeta potential titration curve was measured by an electrokinetic analyzer (SurPASS, Anton Paar GmbH, Graz, Austria) for both functionalized and non-functionalized samples (Ti_Ca+I+TPH and Ti_Ca+I). The zeta potential was determined as a function of pH in an electrolyte solution of 0.001 M KCl, and the pH value (starting at approximately 5.5) was varied by adding 0.05 M HCl or 0.05 M NaOH using the automatic titration unit of the instrument. The isoelectric point (IEP) was established as the intercept of the titration curve with the *x*-axis (zeta potential = 0 mV).

### 2.6. Ion Release

To measure the release of iodine from Ti_Ca+I and Ti_Ca+I+TPH samples, the mentioned samples were immersed in 2 mL of PBS under continuous shaking (50 rpm) at 36.5 °C in the dark. At the planned time points (1 h, 6 h, 24 h, 5 days, and 1 week), the concentrations of iodine ions were measured by inductively coupled plasma emission spectroscopy (ICP, SPS3100, Seiko Instruments Inc., Chiba, Japan). The measurement was performed in triplicate and average and standard deviations were calculated.

### 2.7. Hydroxyapatite Formation

Ti_Ca+I+TPH and Ti_Ca+I were soaked in 24 mL of SBF with ion concentrations close to those of human blood plasma, according to the work of Kokubo [34], for 1 day, 3 days, and 1 week at 36.5 °C. After these time points, the SBF was removed and apatite formation on the substrate was observed with FE-SEM.

### 2.8. SEM

The surfaces of the prepared titanium samples soaked in SBF, Ti_Ca+I, and Ti_Ca+I+TPH were observed under a scanning electron microscope (FE-SEM, S-4300, Hitachi Co., Tokyo, Japan) at an acceleration voltage of 15 kV.

### 2.9. Fluorescence Microscopy

To observe the distribution of surface-grafted polyphenols, their fluorescent properties were exploited [45]. To observe the red fluorescence, a confocal microscope (LSM 900, ZEISS) with a red filter and an excitation wavelength of 573 nm with a 1-s exposure time and a magnification of 100× was used. The test was used to observe the surfaces of Ti_Ca+I and Ti_Ca+I+TPH.

### 2.10. Spectrophotometric Analysis

A modified Folin–Ciocalteu method was used to verify and evaluate the amount of polyphenol grafted onto the surface samples (Ti_Ca+I+TPH and Ti_Ca+TPH). The samples were soaked for 2 h in the dark in a solution with 8 mL of water, 0.5 mL of Folin–Ciocalteu reagent, and 1.5 mL of 20% (*w*/*v*) Na_2_CO_3_ solution [36]. After this time, photometric measurements were performed at 760 nm. To quantify the polyphenol amount, a standard calibration curve was previously prepared using solutions with different gallic acid concentrations (i.e., 0.0025, 0.005, 0.01, 0.02, 0.03, and 0.04 mg/mL) as described in [37]. The Folin–Ciocalteu method quantifies the total phenol content in gallic acid equivalents (GAE) obtained considering a standard calibration curve of gallic acid as a standard for polyphenols’ redox activity [46]. In addition, a second reference curve was cosntructed with defined concentrations of TPH in order to compare the standard GAE values with the concentration values of the specific polyphenolic mixture used in the present work.

### 2.11. Antibacterial Activity Test

The antibacterial activity of untreated titanium (Ti), Ti_Ca+TPH, Ti_Ca+I, and Ti_Ca+I+TPH against *Escherichia coli* (*E. coli*; IFO3972) was estimated according to the ISO22196 standard [38]. Drops of bacterial cell suspensions (100 µL) in RPMI 1640 t broth were injected onto the 25 × 25 × 1 mm^3^-sized samples and covered with a 20 × 20 mm^2^ sterile flexible polypropylene film so that the solutions were firmly in contact with the substrate. The samples thus prepared were stored in Petri dishes in an incubator with controlled humidity and temperature (95% and 35 °C, respectively) for 24 h. After 24 h, the samples were removed from the incubator and rinsed with 10 mL of soybean casein digestion bar containing lecithin and polyoxyethylenesorbitan monooleate (SCDLP) to collect bacteria grown during incubation. Finally, the number of bacterial cells was calculated using the dilution factor and the number of colonies counted on the Petri dish.

### 2.12. Cytocompatibility Evaluation

Biological characterizations were performed using square specimens (10 mm × 10 mm × 1 mm) sterilized by UV light (2 h). Specimen cytocompatibility was tested in vitro towards human bone marrow-derived stem cells (hMSC); cells were obtained from Merck (Promo Cell C-12974) and cultivated in low-glucose Dulbecco’s modified Eagle Medium (DMEM, Sigma Aldrich, Milan, Italy) supplemented with 15% fetal bovine serum (FBS, Sigma Aldrich, Milan, Italy) and 1% antibiotics (penicillin/streptomycin) at 37 °C and in a 5% CO_2_ atmosphere. Cells were cultivated until 80–90% confluence, detached by a trypsin-EDTA solution (0.25% in PBS), harvested, and then used for the experiments.

For cytocompatibility studies, specimens were gently placed into a p24 multi-well plate and cells were seeded dropwise with a specific number of cells (1 × 10^4^ cells/specimen) onto the specimens’ surface and allowed to adhere for 4 h at 37 °C before being submerged in 1 mL of medium. After 24 h of cultivation in direct contact with the specimen surface, the cell viability was evaluated by their metabolic activity by the colorimetric metabolic Alamar blue assay (AlamarBlue™, ready-to-use solution from Life Technologies, Milan, Italy). Briefly, after introducing the Alamar solution, the plate was incubated in the dark for 4 h at 37 °C; then, the supernatants were collected and the fluorescence signals were evaluated with a spectrophotometer (Spark^®^, Tecan Trading AG, Zürich, Switzerland) using the following set-up: fluorescence excitation wavelength 570 nm and fluorescence emission reading 590 nm. Moreover, cells adhered to the specimen surface were investigated for their viability by the fluorescent live/dead assay (LIVE/DEAD, from Invitrogen, Milan, Italy); briefly, after washing with PBS, the solution was added to each specimen and then incubated for 45 min. After incubation, the specimens were washed with PBS and fluorescent images were collected by a confocal microscope (Leica SP8 confocal platform, Leica Microsystems, Germany).

### 2.13. Antioxidant Properties Evaluation

The specimens’ ability to act as an antioxidant by scavenger process was evaluated regarding their ability to promote cell survival in a pro-inflammatory environment. Accordingly, the inflammation was chemically induced by adding hydrogen peroxide (H_2_O_2_, 3 h, 300 mM) into the medium to generate oxidative stress by toxic active species as previously shown by the authors in [47]. Accordingly, H_2_O_2_ was added before the cell seeding to resemble a pre-implant inflammation, or after the seeding to simulate a post-operative inflammation, as fully detailed in Supplementary Files S1 and S2, respectively. Afterwards, the cell metabolic activity was evaluated by the Alamar blue assay as previously detailed. Moreover, to demonstrate that the toxic effect was due to the internalization of toxic active species, the specific CellRox reagent (CellROX™ Deep Red Reagent kit, from Thermo Fisher Scientific, Milan, Italy) was used to visualize the species in the intracellular compartment; cells were further co-stained with phalloidin (Alexa Fluor 488 Phalloidin, from Thermo Fisher Scientific, Milan, Italy) and 4,6-diamidino-2-phenylindole (DAPI, Sigma Aldrich, Milan, Italy) to visualize cytoskeleton F-actin filaments and nuclei, respectively. Cells cultivated in a regular medium were considered a positive control.

## 3. Results and Discussion

The first part of the research was devoted to verifying the effective presence of both polyphenols and iodine ions on the functionalized surfaces and to their characterization. The Ti_Ca+I surface has already been characterized in [19] and it was here used as a reference.

Table 1 shows the atomic percentages of the elements, as detected by XPS, on the surfaces before and after functionalization. The functionalized samples show a reduction in the atomic percentage of titanium with respect to Ti_Ca+I, evidencing that a layer, attributable to polyphenols, covers the titanium oxide surface. In addition, the functionalized samples (Ti_Ca+I+TPH) show a 60% reduction in the atomic percentage of iodine. One explanation of this effect is that the organic molecules covered the surface, thus also covering the surface-exposed iodine ions as was the case for titanium. Another explanation could be that the functionalization method led to an ionic release at the interface between the sample surface and the drop of the polyphenol solution, thus leading to a decrease in iodine on the functionalized surface. The ratio between the titanium and iodine percentages was similar on both surfaces (21 on TiCa+I+TPH and 18 on Ti_Ca+I), suggesting that the first explanation was more appropriate and that iodine release during the functionalization was quite limited.

Confirming this, the analysis survey also shows an important increase in the percentage of carbon, the main element that composes polyphenols. Oxygen, on the other hand, does not change drastically because the polyphenols, being OH-rich, exposed the oxygen on the functionalized surface as was the case for titanium oxide before functionalization. Lastly, both surfaces contain calcium. This can be explained considering that the chemical treatment used for obtaining Ti_Ca+I induced a calcium enrichment in the titanium oxide layer, as well as by considering the functionalization with polyphenols was performed in a solution with Ca^2+^ ions.

**Figure 2:**
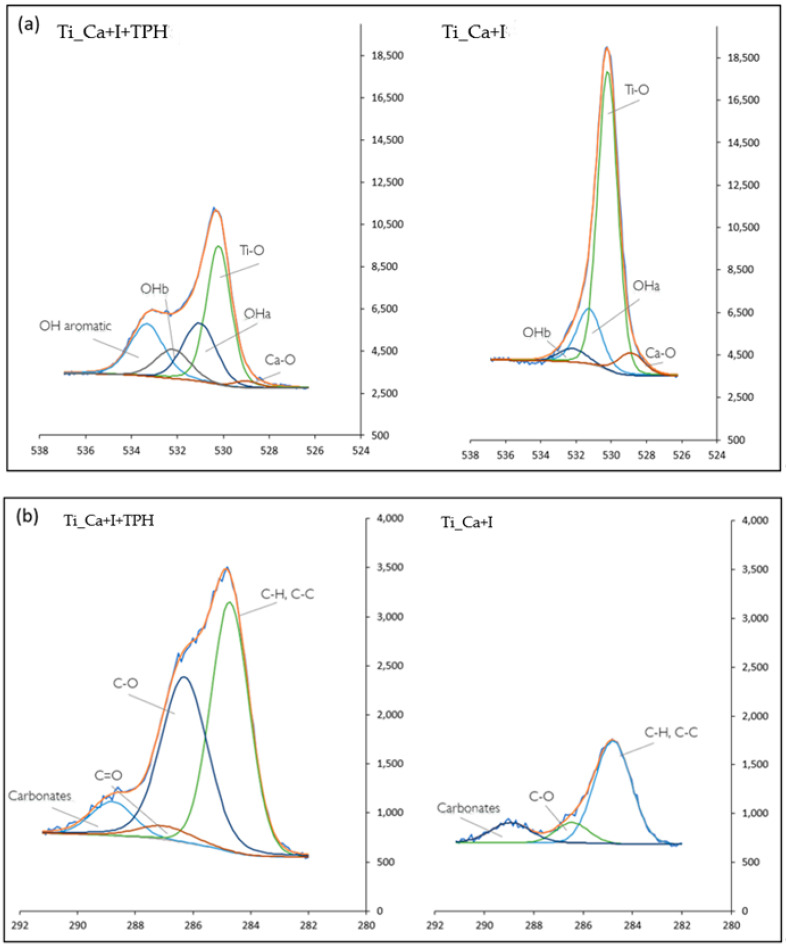
XPS high resolution (HR) spectra of (**a**) O1s and (**b**) C1s of the samples Ti_Ca+I+TPH and Ti_Ca+I.

To identify the specific chemical groups exposed on the surfaces, high-resolution spectra of carbon and oxygen (C1s and O1s) were measured, as shown in Figure 2, which represent the main molecules that characterize polyphenols.

After TPH grafting, different contributions were detected in the carbon region (Figure 2b). In particular, although the peak of C-C and C-H bonds at 284.7 was the highest in both cases, the peak related to C-O at 287.5 eV [48] became higher after functionalization, as expected when polyphenols are compared to adventitious organic contaminations (mainly hydrocarbon). Moreover, a new signal can be observed at about 288 eV. This can be attributed to C=O bonds, already detected on surfaces functionalized with polyphenols [43] and explained by partial oxidation of the molecules creating a quinone bond. The presence of a peak due to carbonates can be due to some impurities.

In the oxygen region, the difference between the functionalized and non-functionalized samples was clearer and more visible (Figure 2a). Three peaks were observable on the Ti_Ca+I sample at 530.44 eV, 531.5, and 532.5 eV and can be correlated with the Ti-O bond of the titanium oxide, with the –OH acid (OH_a_), and with the basic (OH_b_) groups exposed by the surface titanium oxide layer after the treatment, respectively [22,49]. In the spectrum of the Ti_Ca+I+TPH sample, these peaks are still observable, but a new peak is visible at 533.8 eV, related to the OH aromatic band that is attributable to polyphenols [49]. It is of interest that the ratio between the area of the peaks related to OH_b_ and OH_a_ was almost the same before and after functionalization (OH_b_/OH_a_ was 3 after functionalization and 4 before it), confirming that these functional groups belonged to the titanium oxide layer. Analogously, the ratio between the area of the peak related to TiO and the sum of the peaks related to OH_b_ and OH_a_ did not significantly change before and after functionalization (TiO/(OH_a_+OH_b_) was 0.4 after functionalization and it was 0.3 before it). Both these ratios, shown in Table 2, confirmed that the OH groups of the polyphenols were related to the phenolic signal while the others belonged to the titanium oxide layer. In both cases, a signal related to Ca-O was present, this was due to the calcium titanate in Ti_Ca+I and to the Ca^2+^ ions linking the polyphenols in Ti_Ca+I+TPH.

**Figure 3:**
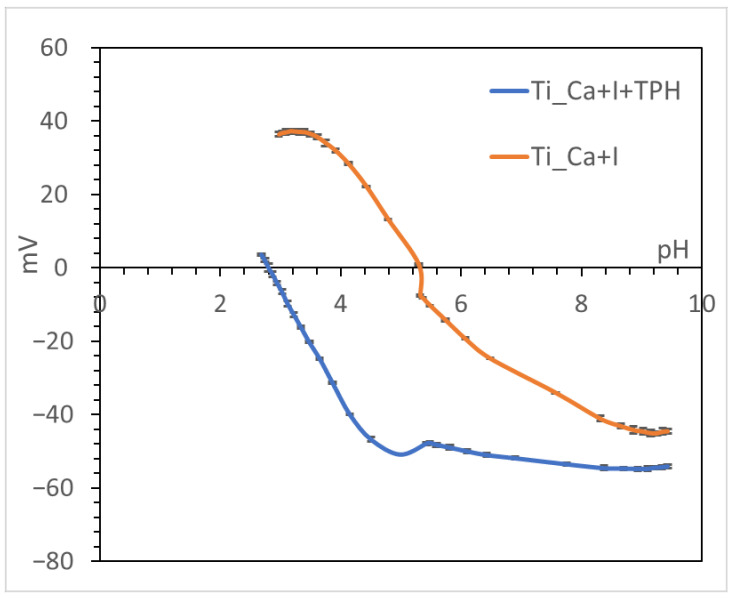
Zeta potential titration curve for Ti_Ca+I and Ti_Ca+I+TPH.

Figure 3 shows the zeta potential titration curves of titanium samples with (Ti_Ca+I+TPH) and without polyphenols (Ti_Ca+I). The isoelectric point (IEP) of the surface before functionalization was about 5.3 and the surface was negatively charged at physiological pH (about −34 mV at pH = 7.4). The curve shows an evident plateau in the acidic range (with positive zeta potential values), which can be explained by the presence of basic functional groups (supposedly basic OH). The beginning of the acidic plateau was at pH ≈ 4, implying that the functional groups behaved as a very strong base; in fact, they protonated at a high pH value. On the other hand, the surface was also rich in acid functionalities (presumably OH acid groups), confirmed by the presence of a plateau in the basic range with an onset of pH 8.5. In fact, surface treatment, in addition to creating basic groups, created some acidic groups, but they acted as a weak acid and were deprotonated only at very high pH. The prevalence of the basic groups in determining the surface charge and zeta potential, because of the stronger reactivity, was evidenced by a shift in the IEP towards a higher value (5.3) with respect to untreated titanium (expected IEP at pH 4). A contribution by basic and acidic functionalities due to iodine (HOI as an amphoteric functional group) may also be supposed [50].

On the other hand, looking at the curve of the functionalized sample (Ti_Ca+I+TPH), the shift in the isoelectric point toward the acidic range and the disappearance of the plateau at acidic pH can be explained by the polyphenols covering the surface and the basic functional groups of Ti_Ca+I, thus exposing many more acidic OHs characteristic of the polyphenol molecules. This fact also led to the appearance of a much more pronounced plateau from a pH value of 4.5, indicating that the acidic OHs behaved as a strong acid. This is expected for some of the OH groups of polyphenols (EGCG, in particular) which have a pKa value lower than 7 [51].

The standard deviation of the zeta potential was very small for all measurements (the error bars are hardly visible in the graph), both for the reference and functionalized sample, demonstrating the good surface chemical stability [52] and the efficacy of polyphenol grafting on the surface.

Looking at the XPS data and comparing them with the zeta potential curves, we can say with certainty that the acidic and basic functional properties of the Ti_Ca+I samples were promoted by the basic and acidic OH groups exposed on the surface as a result of the treatment.

**Figure 4:**
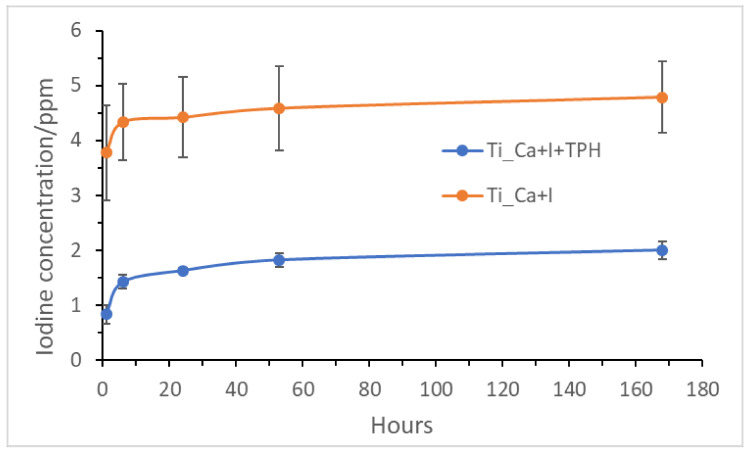
Iodine concentration released from Ti_Ca+I and Ti_Ca+I+TPH as a function of the soaking time in PBS (in hours).

Figure 4 shows the concentration of iodine released from the treated Ti_Ca+I and Ti_Ca+I+TPH samples as a function of immersion time in PBS, measured by inductively coupled plasma emission spectroscopy.

The graph shows that the treated Ti_Ca+I initially released 4.0 ppm of iodine within 6 h and then slowly released another 1.6 ppm over one week. In contrast, the functionalized sample (Ti_Ca+I+TPH) shows a significant decrease in ionic release, about half the amount of the reference sample, indicating that the functionalization mode probably reduces the kinetics of the ion release and slightly reduces the amount of iodine available on the sample surface, as confirmed by XPS. Interestingly, the presence of polyphenols does not alter the mechanism of ion release; the release trend remains the same even with a lower absolute value in terms of concentration. The standard deviation was also lowered after the functionalization, evidencing a lower but probably more stable release.

**Figure 5:**
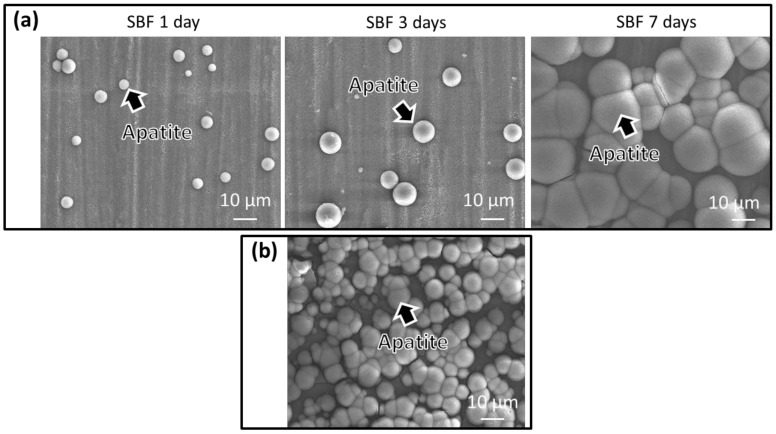
FE-SEM images of the surfaces soaked in SBF of (**a**) Ti_Ca+I+TPH for 1, 3, and 7 days, (**b**) Ti_Ca+I for 3 days.

Figure 5a shows the formation of apatite on the functionalized samples (Ti_Ca+I+TPH) after 1, 3, and 7 days. Apatite formation on the surface of an implant is a key factor in determining whether an implant is osteoconductive and can be integrated with the surrounding bone tissue. It is important because it creates a surface similar to the mineral structure of the natural bone. This allows for better osseointegration, which is essential for the long-term stability and success of the implant [53,54].

After immersion in SBF, numerous spherical particles were formed on the sample surface, which by day 7 covered almost the entire surface. In contrast, Figure 5b shows the formation of apatite on a reference sample (Ti_Ca+I) after 3 days. Polyphenols seem to slow the growth of hydroxyapatite, as it was clear that after 3 days fewer and smaller particles were found on the functionalized sample. In fact, in general, polyphenols interact with hydroxyapatite by binding to the surface of the apatite crystals. This binding can affect the nucleation and growth of the apatite crystals, leading to changes in the size and shape of the crystals [55,56]. In any case, the surfaces had a remarkable ability to induce hydroxyapatite precipitation during soaking in simulated body fluid, as it appeared after 7 days of soaking.

**Figure 6:**
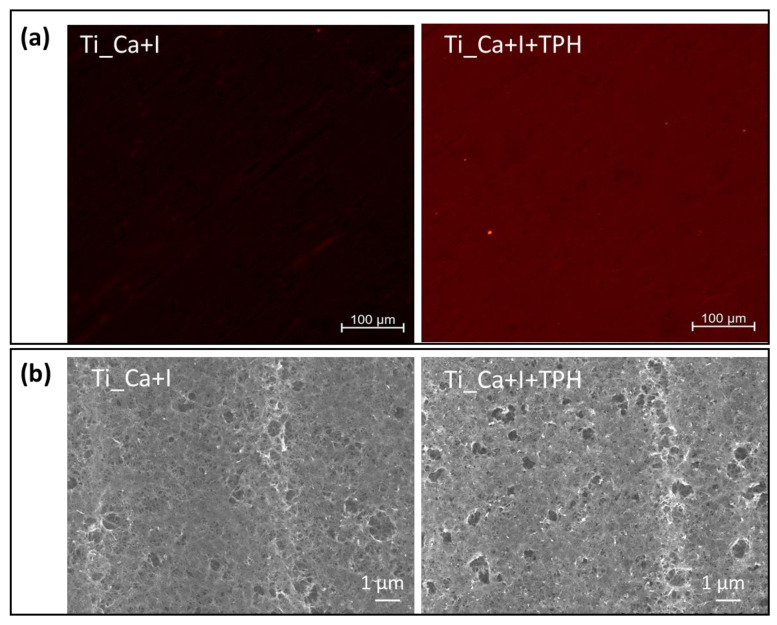
Morphology of Ti_Ca+I and Ti_Ca+I+TPH samples. (**a**) Fluorescence microscopy observations.(**b**) Surface FE-SEM images.

The distribution of polyphenols on the sample surface was studied by exploiting the autofluorescent abilities of polyphenols. Figure 6a shows the fluorescence microscope observations of the Ti_Ca+I sample before and after functionalization. On the surface of the reference sample (Ti_Ca+I), the fluorescent signal was absent, confirming that the surface itself is not fluorescent. On the other hand, the surface of the functionalized sample (Ti_Ca+I+TPH) showed a uniform intensity of the emitted fluorescent signal, which means that the grafted polyphenols were characterized by a uniform distribution on the surface of the material, forming a thin but homogeneous layer. It is also important to note from the SEM images of the functionalized and reference surfaces, Ti_Ca+I-TPH and Ti_Ca+I, respectively (Figure 6b), that although polyphenols were uniformly present on the sample surface, they did not alter the nanotextural morphology created by the treatment, which is important for osseointegration.

**Figure 7:**
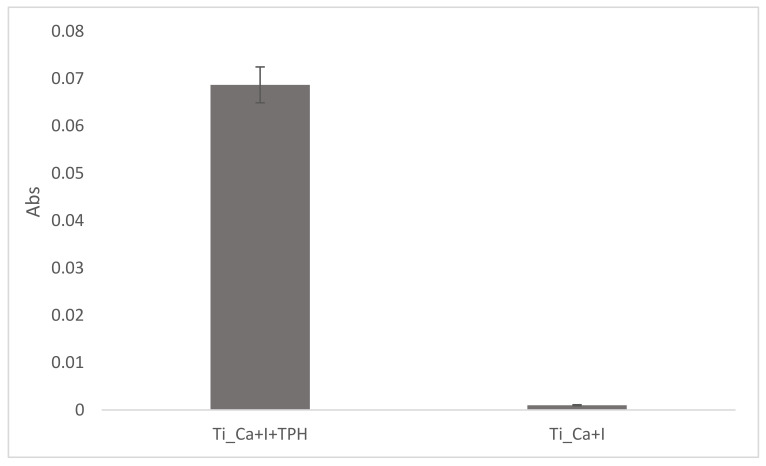
The redox activity in absorbance of polyphenols grafted on the surfaces, measured through the modified Folin–Ciocalteu method, of Ti_Ca+I+TPH and Ti_Ca+I samples.

Quantification of surface polyphenols, on the other hand, was studied by exploiting the antioxidant capacity of polyphenols using spectrophotometric methods such as the modified Folin–Ciocolteu analysis test. As shown in Figure 7, the absorbance at 760 nm was around 0.07, evidencing a redox activity of surface polyphenols equivalent to a solution with a gallic acid concentration of 0.0030 mg/mL (GAE). This result evidenced that the grafted polyphenols maintain their redox chemical activity after grafting. The Folin–Ciocalteu method uses a calibration curve obtained with gallic acid. Using a similar reference curve obtained by using TPH, an absorbance of 0.07 corresponds to a concentration of TPH of 0.0038 mg/mL, which is close to the one obtained in GAE (by using the calibration curve with gallic acid). According to a previous work of the authors [44], the CT surface has a limited amount of active sites for functionalization and they are saturated by functionalization with a gallic acid solution of 1 mg/mL (the analogies between GA and TPH calibration curves were supported the comparison of the two functionalization routes).

**Figure 8:**
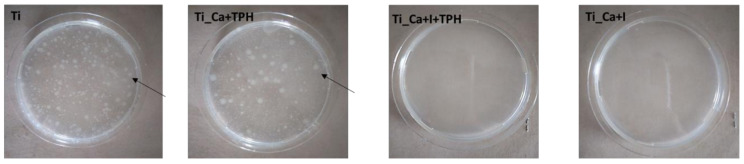
Optical images of colony formation on Ti, Ti_Ca+TPH, Ti_Ca+I+TPH, and Ti_Ca+I.

The antibacterial effect of iodine is well known and has been already studied in previous works [22]. An antibacterial test was carried out according to the ISO22196 standard on Ti, Ti_Ca+TPH, Ti_Ca_I+TPH, and Ti_Ca_I samples. A larger set of samples has been used for this test to understand whether the presence of polyphenols affected the antibacterial effect of iodine. Accordingly, the specimen Ti_Ca+THP was included in the control groups of the antibacterial studies, beyond the untreated Ti substrate, to verify a possible contribution of the polyphenols in reducing bacterial infection on the multifunctional surface. Figure 8 shows that the functionalized and reference samples, without iodine ions on the surface (Ti and Ti_Ca+TPH), have no kind of antibacterial effect against *E. coli*, being free of the antibacterial agent. It is also evident that the presence of polyphenols on the surface did not inhibit bacterial colonization and did not show any antibacterial effect. Samples with iodine on the surface (Ti_Ca+I and Ti_Ca+I+TPH), on the other hand, did not show colony formation either before or after functionalization, indicating a potent antibacterial activity of iodine on *E. coli* (with a percentage reduction of >99%), as shown in Table 3. Therefore, the presence of polyphenols did not inhibit the antibacterial capacity of iodine, which remained intact, even if the ion release was reduced.

To exclude any toxic effect, cytocompatibility was verified before moving to the antioxidant property evaluation. Given the potential application of the here developed specimens for bone repair, human mesenchymal stem cells (hMSC) were considered as a target model in view of their pivotal role in the healing process. Moreover, cells were seeded directly in direct contact with the control and functionalized surfaces to study their ability to properly adhere, spread, and exploit metabolic activity, thus simulating possible colonization of the device after implantation by the cells recruited from the neighbor tissue towards the injury site [57].

**Figure 9:**
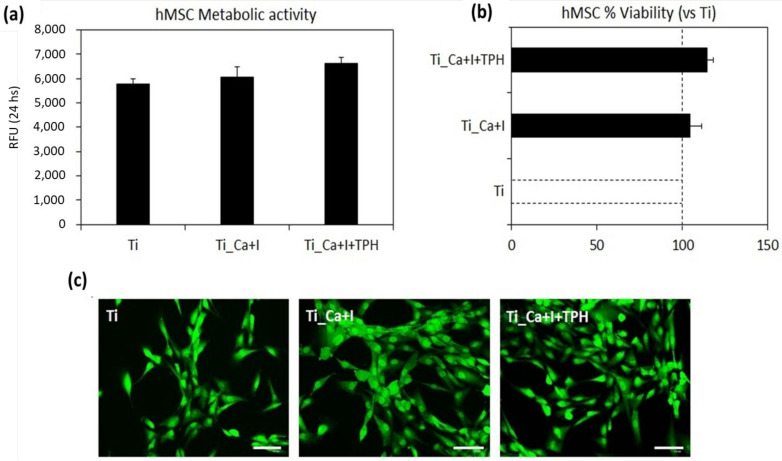
Cytocompatibility evaluation. Surface functionalization with iodine (Ti_Ca+I) and polyphenols (Ti_Ca+I+TPH) did not decrease hMSC metabolic activity in comparison to the Ti controls ((**a**), *p* > 0.05) giving a positive boost of ≈5% and ≈15%, respectively. (**c**), Live/dead images confirmed that cells were viable (stained in green) with a proper morphology and comparable surface density. (**b**), bars represent means ± dev.st, replicates = 3. Images bar scale = 125 μm.

Metabolic activity was considered as the parameter to determine the viability of the cells adhered to the specimen surface after 24 h of cultivation, with the results reported in Figure 9. Neither of the treated surfaces (Ti_Ca+I or Ti_Ca+I+TPH) showed a reduction in the hMSC metabolic activity in comparison to the untreated Ti control (*p* > 0.05), that was considered as a positive control given the large literature demonstrating its biocompatibility [58]. On the contrary, the presence of polyphenols (TPH) lead to an increase (≈15%) in the metabolism in comparison to the Ti control that was assumed as 100% as discussed prior (Figure 9b). It can be hypothesized that the bioactivity of the functionalized specimens was improved; thus, they are hypothetically able to improve/speed up the healing process. In fact, the previous literature demonstrates that green tea polyphenols can improve or increase the speed of different mechanisms related to mesenchymal stem cells, such as proliferation and differentiation [59] or osteogenesis [60], by different molecular pathways, as reviewed by Trzeciakiewicz et al. [61] when investigating bone physiology. Therefore, the polyphenols applied here from green tea demonstrated an interesting pro-regenerative potential similar to other well-known polyphenols, such as resveratrol [62], which stimulates hMSC to release growth factors in non-healing wounds, and curcumin, which improves BMP-2 and TGF-β production ameliorating osteogenesis in a pro-inflammatory environment [63]. On the contrary, the iodine contribution appeared insignificant in terms of the hMSC metabolism boost (≈5% improvement in comparison to Ti control); however, no previous research has reported a significant pro-regenerative or pro-healing activity of iodine towards stem cells. It is mostly recognized as a strong antioxidant compound [64] as well as a potential antibacterial agent, as also previously demonstrated in this work. To obtain a visual confirmation of the specimens’ successful colonization, live/dead fluorescent imaging (Figure 9c) was applied to confirm that cells were viable (stained in green) showing a correct fibroblast-like morphology and a comparable surface density between the control and functionalized samples.

We imposed an oxidative stress to the hMSC seeded onto the surface of the control (Ti) and functionalized specimens (Ti_Ca+I and Ti_Ca+I+TPH) by the generation of active species through H_2_O_2_ medium doping, to verify if any scavenger action was performed by the surfaces. In the first experimental set-up, a pro-inflammatory environment was induced before cell seeding to verify if the functionalization was effective in reducing the amount of free toxic active species, affecting the cells migrating towards the implant site. The protocol was first validated using polystyrene as a gold standard substrate for cell cultivation and the results are reported in Supplementary Figure S1.

**Figure 10:**
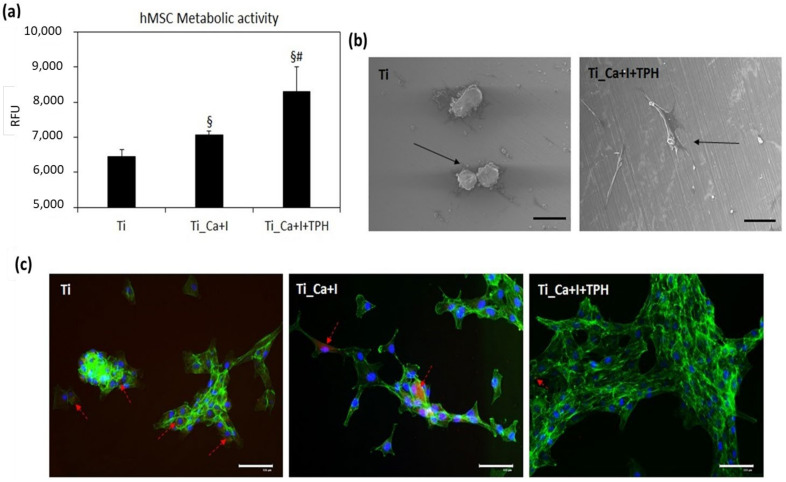
Pre-inflammatory evaluation. When the inflammatory environment was generated before cell seeding, the presence of iodine + polyphenols (Ti_Ca+I+TPH) protected cells from oxidative stress, as they showed the highest metabolism ((**a**), *p* < 0.05 in comparison with Ti and Ti_Ca+I, indicated by § and #, respectively) and a proper morphology in SEM images (**b**). The toxic effect was due to the active species internalization, as shown in the CellRox staining ((**c**), positive cells stained in red) where most of the resulting cells were protected by the iodine and polyphenol. Bars represent means ± st. dev, replicates = 3. SEM images: 180× magnification, bar scale = 100 μm; fluorescent images bar scale = 125 μm.

Results related to the specimen tests are reported in Figure 10. As expected, the lowest value in terms of cell metabolic activity was registered by the untreated Ti controls (Figure 10a); the lack of any protective effect was determined by a toxic environment driving cells mostly to the apoptotic stage, as clearly seen by the SEM images (Figure 10b), where cells appeared mostly in the typical apoptotic round shape. On the contrary, iodine (Ti_Ca+I) provided a first protection showing a significant improvement in comparison to the Ti control (Figure 10a, *p* < 0.05 indicated by §), but the best results were achieved by the combination of iodine + polyphenol (Ti_Ca+I+TPH), with changes that were significant in comparison to both the control and iodine (Figure 10a, *p* < 0.05 indicated by § and #, respectively). SEM images (Figure 10b) confirmed that cells seeded onto the functionalized surfaces were able to adhere, spread, and maintain a proper fibroblast-like morphology. Their metabolism was also the highest when the Alamar blue assay was applied. Therefore, it can be hypothesized that iodine and polyphenols worked in synergy to maximize the capture of the active species, thus protecting cells from their toxic effect. To confirm this hypothesis, the specific fluorescent assay CellRox was applied to visualize the active species internalized by the cells. As displayed in Figure 10c, most of the cells cultivated onto Ti were positive to the red color of the dye (indicated by the red arrows), whereas the number of cells positive decreased when iodine and iodine/polyphenols surfaces were analyzed. Moreover, as previously observed by SEM, cells on the Ti controls displayed mostly a round-shaped morphology, forming clusters. On the contary, cells cultivated in the Ti_Ca+I+TPH specimens showed the highest density, a proper morphology, and the lowest number of red-positive signals, thus giving a clearly confirming the starting hypothesis.

In the second experimental set-up, the pro-inflammatory environment was induced after cell seeding to verify if the functionalization was effective in protecting from free toxic active species where the cells colonized the implant site. The protocol was first validated using polystyrene as a gold standard substrate for cell cultivation and the results are reported in Supplementary Figure S2.

**Figure 11:**
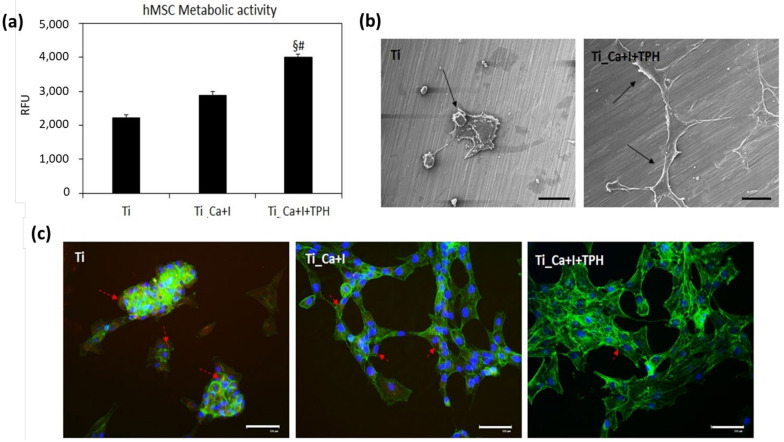
Pro-inflammatory evaluation. When the inflammatory environment was generated after cell seeding, the presence of iodine + polyphenols (Ti_Ca+I+TPH) protected cells from oxidative stress, as the highest metabolism was highest ((**a**), *p* < 0.05 in comparison with Ti and Ti_Ca+I, indicated by § and #, respectively), and a proper morphology in the SEM images (**b**). The toxic effect was due to the active species internalization as shown in the CellRox staining ((**c**), positive cells stained in red), where most of the cells were protected by the iodine and polyphenol. Bars represent means ± st. dev, replicates = 3. SEM images: 180× magnification, bar scale = 100 μm; fluorescent images bar scale = 125 μm.

In a very similar way to the first experimental set-up, the lowest value in terms of cell metabolic activity was reported by the untreated Ti controls (Figure 11a); however, in this second pro-inflammatory condition only the functionalization based on the iodine + polyphenols reported significant results in comparison to both Ti controls and iodine functionalized Ti_Ca+I (Figure 11a, *p* < 0.05 indicated by § and #, respectively). Probably, the presence of the cells seeded before H_2_O_2_ administration reduced the scavenger activity of the surfaces by “screening” some of the surface-exposed iodine ions and polyphenols; therefore, only the combination was effective in protecting the cells from oxidative stress. Therefore, the very promising protective effect of the iodine + polyphenols combination was confirmed, as the cells maintained the correct morphology, as seen by the SEM images (Figure 11b), and minimized the toxic effect of the active species internalization, as confirmed by the CellRox fluorescent dye (Figure 11c). This assay was specifically chosen considering the hypothesis that TPH can act as a scavenger, thus preventing the intracellular accumulation of toxic active species generated by the H_2_O_2_ administration.

The results agree with the literature. Iodine is largely demonstrated to have an intrinsic antioxidant activity that can be exploited to reduce inflammation in situ upon implantation for tissue engineering purposes. In fact, iodine is internalized by a facilitated diffusion system that is conserved and it directly neutralizes free radicals, induces the expression of type II antioxidant enzymes, or inactivates proinflammatory pathways [65]. Moreover, here, iodine was combined with polyphenols, another element conferring strong antioxidant activity to the functionalized surfaces, as previously demonstrated also by the authors [65,66]. In fact, polyphenols are very promising chemicals as they improve tissue repair under pro-inflammatory conditions. Regarding bone repair, polyphenols stimulate bone formation, mineralization, proliferation, differentiation, and the survival of osteoblasts by a protective effect against oxidative stress and inflammatory cytokines [67]. In addition, polyphenols inhibit the differentiation of the osteoclast cells, thus favoring bone repair over resorption [67].

The exploitation of the combination of TPH + iodine confirmed the starting hypothesis that this combination confers antioxidant properties to the ion-doped surface. Future further studies are still necessary to understand the cell response in terms of up- or down-regulation of pro- and anti-inflammatory markers to better define in which pathways THP and iodine are involved.

## 4. Conclusions

Green tea polyphenols were successfully grafted onto the surface of chemically treated titanium doped with iodine ions. Physicochemical characterization was performed, and the presence, amount, distribution, and release of the grafted polyphenols were evaluated. The reported results showed a stable molecular grafting, uniform on the surface, which allowed hydroxyapatite precipitation. The polyphenols showed a good antioxidant effect in the biological environment and did not alter the antibacterial effect of iodine or the morphology of the surface, thus succeeding in obtaining a multifunctional surface with anti-inflammatory, antibacterial, and osseointegration capabilities. Cell assays confirmed that the functionalization was cytocompatible towards human mesenchymal stem cells and the combination of iodine and polyphenols demonstrated a strong ability to protect cells from chemically induced oxidative stress, thus representing a promising strategy to provide support in the healing process under pro-inflammatory conditions.

## Figures and Tables

**Table 1:** The atomic percentages of the elements detected on the surface of samples Ti_Ca+I+TPH and Ti_Ca+I.

Atomic Percentage					
	C1s	O1s	Ca2p	Ti2p	I3d5
**Ti_Ca+I+TPH**	41.24	45.28	1.75	11.2	0.53
**Ti_Ca+I**	13.79	59.49	1.57	23.79	1.36

**Table 2:** The ratio of the peak area of the XPS curves for the Ti_Ca+I+TPH and Ti_Ca+I samples.

	OH_a_/OH_b_	TiO/(OH_a_+OH_b_)
Ti_Ca+I+TPH	3	0.4
Ti_Ca+I	4	0.3

**Table 3:** Antibacterial activity values of S1_Ti against *E. coli*.

Average of *E. coli* Count/CFU			
Ti	Ti_Ca+TPH	Ti_Ca+I+TPH	Ti_Ca+I
9.40 × 10^6^	5.07 × 10^6^	<20	<20

## Data Availability

No new sharable data were created.

## Supplementary Materials

The following supporting information can be downloaded at: https://www.mdpi.com/article/10.3390/nano13030479/s1, Figure S1. The use of H_2_O_2_ to pre-induce the formation of an inflammatory environment (a) determined a significant reduction of the cells’ metabolism in comparison to the untreated controls ((b), *p* < 0.05 indicated by §). As a confirmation, toxic active species were detected in the majority of the cells exposed to such pre-conditioned environment ((c), positive cells stained in red by CellRox dye). Bars represent means ± dev.st, replicates = 3. Images bar scale = 125 μm; Figure S2: The use of H_2_O_2_ to generate toxic active species resembling an inflammatory environment (a) determined a significant reduction of the cells’ metabolism in comparison to the untreated controls ((b), *p* < 0.05 indicated by §). As a confirmation, toxic active species were detected in the majority of the cells exposed to H_2_O_2_-doped medium ((c), positive cells stained in red by CellRox dye). Bars represent means ± dev.st, replicates = 3. Images bar scale = 125 μm.
