# Supplementary material for: Conferring Antioxidant Activity to an Antibacterial and Bioactive Titanium Surface through the Grafting of a Natural Extract
Source: Nanomaterials (Basel). 2023 Jan 25;13(3):479. doi: 10.3390/nano13030479 (PMC9919197; doi:10.3390/nano13030479)
Supplement: Supplementary file 1 [file nanomaterials-13-00479-s001.zip › nanomaterials-2147349-supplementary.pdf]

Conferring Antioxidant Activity to an Antibacterial and Bioactive Titanium Surface through the Grafting of a Natural Extract

Francesca Gamna [1,*], Seiji Yamaguchi [2], Andrea Cochis [3], Sara Ferraris [1], Ajay. Kumar [3], Lia Rimondini [3] and Silvia Spriano [1,*]

[1] Politecnico di Torino, Corso Duca degli Abruzzi 24, Corso Duca degli Abruzzi 24, 10129 Torino, Italy;

[2] Department of Biomedical Sciences, College of Life and Health Sciences, Chubu University, 1200 Matsumoto, Kasugai 487-8501, Aichi, Japan;

[3] Department of Health Sciences, Center for Translational Research on Autoimmune and Allergic Diseases − CAAD, Università Del Piemonte Orientale UPO, Corso Trieste 15/A, Novara (NO), 28100, Italy

Supplementary Information

S1. Validation of the pre-inflammatory model in polystyrene

Prior to apply the pre-inflammatory protocol to the control e treated Ti specimens, it was pre-validated by using the polystyrene gold standard as substrate for cells' cultivation. Accordingly, 300 mM H_2O_2 were added into the medium in order to introduce toxic active species; after 3 hours, cells (hMSC, $1x10^4$ cells/specimen) were seeded onto the wells of a 24 multiwell plate and submerged with 1 ml/well of the pre-conditioned medium (schematized in Supplementary Figure 1a). Cells were cultivated for 24 hours and then their metabolic activity was evaluated by the alarm blue assay as detailed in the main paper. Moreover, to demonstrate that the toxic effect was due to the internalization of toxic active species, the specific CellRox reagent (CellROX™ Deep Red Reagent kit, from Thermo Fisher Scientific, Milan, Italy) was used to visualize the species in the intracellular compartment; cells were further co-stained with phalloidin (Alexa Fluor 488 Phalloidin, from Thermo Fisher Scientific, Milan, Italy) and 4,6-diamidino-2-phenylindole (DAPI, Sigma Aldrich, Milan, Italy) to visualize cytoskeleton F-actin filaments and nuclei, respectively. Cells cultivated in regular medium were considered as positive control.

Results are reported in Supplementary Figure 1. As can be noted by the metabolic assay (Supplementary Fig. 1b), the induced pro-inflammatory environment caused a significant reduction of the cell's metabolism (≈54%) being significant in comparison to the untreated controls ($p<0.05$, indicated by §). Therefore, the cells were able to sense the toxic environment reducing their metabolism and most likely their number as can be hypothesized from the density of the DAPI-labelled nuclei. As a confirmation of the cytotoxicity induced by the generated active species, the specific CellRox fluorescent dye was detected in the majority of the cells exposed to the H_2O_2-doped medium, thus giving a confirmation of the suitability of the proposed protocol to resemble a material applied into an inflamed environment.

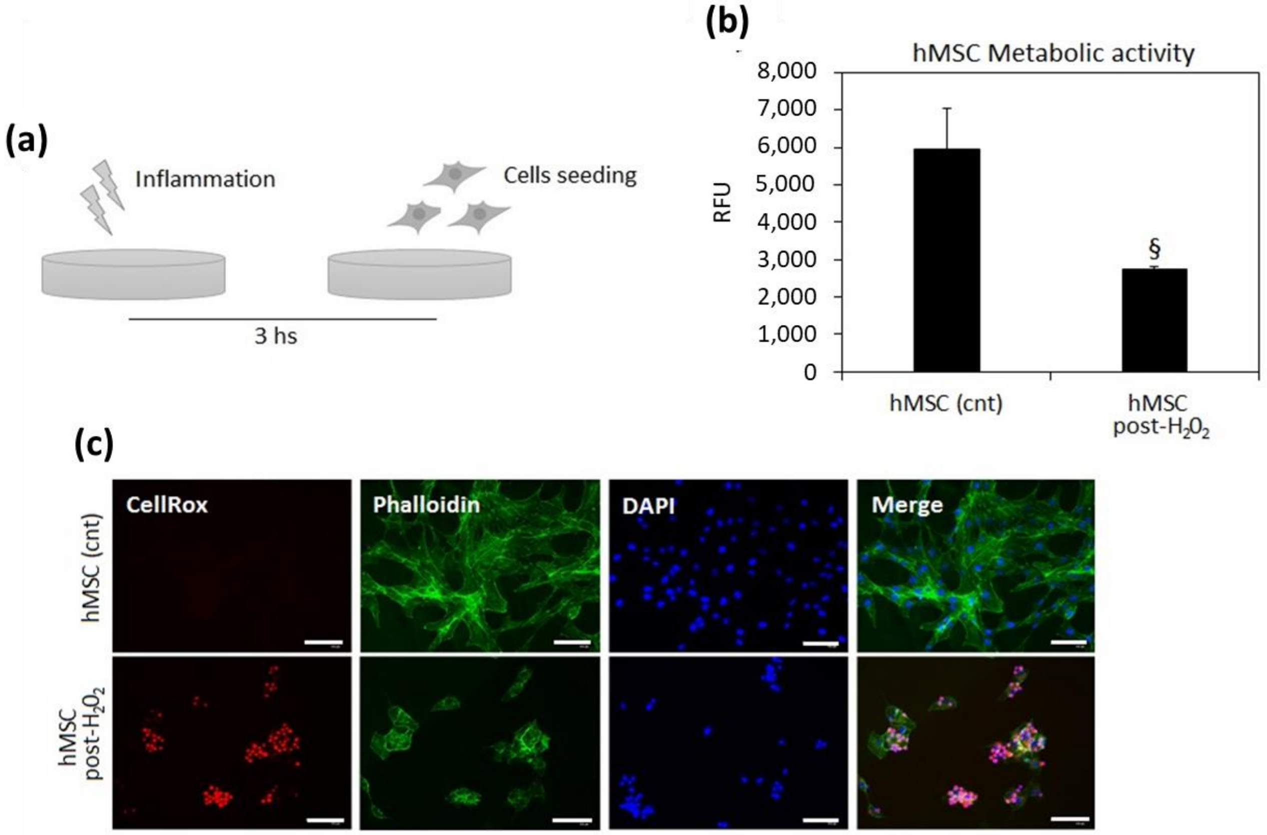

Figure S1*. The use of H_2O_2 to pre-induce the formation of an inflammatory environment (**a**) determined a significant reduction of the cells' metabolism in comparison to the untreated controls ((**b**), p<0.05 indicated by §). As a confirmation, toxic active species were detected in the majority of the cells exposed to such pre-conditioned environment ((**c**), positive cells stained in red by CellRox dye). Bars represent means±dev.st, replicates=3. Images bar scale=125 μm.*

S2. Validation of the pro-inflammatory model in polystyrene

Prior to apply the pro-inflammatory protocol to the control e treated Ti specimens, it was pre-validated by using the polystyrene gold standard as substrate for cells' cultivation. Accordingly, cells (hMSC, $1x10^4$ cells/specimen) were seeded onto the wells of a 24 multiwell plate and submerged with 1 ml/well of medium. After 24 hours, 300 mM H_2O_2 were added into the medium in order to introduce toxic active species (schematized in Supplementary Figure 2a); after 3 hours cells' metabolic activity was evaluated by the alarm blue assay as detailed in the main paper. Moreover, to demonstrate that the toxic effect was due to the internalization of toxic active species, the specific CellRox reagent (CellROX™ Deep Red Reagent kit, from Thermo Fisher Scientific, Milan, Italy) was used to visualize the species in the intracellular compartment; cells were further co-stained with phalloidin (Alexa Fluor 488 Phalloidin, from Thermo Fisher Scientific, Milan, Italy) and 4,6-diamidino-2-phenylindole (DAPI, Sigma Aldrich, Milan, Italy) to visualize cytoskeleton F-actin filaments and nuclei, respectively. Cells cultivated in regular medium were considered as positive control. Results are reported in Supplementary Figure 2. As can be speculated by the metabolic assay (Supplementary Fig. 2b), the introduction of toxic active species by H_2O_2 exposition caused a significant reduction of the cell's metabolism (≈62%) being significant in comparison to the untreated controls ($p<0.05$, indicated by §). Therefore, the cells reacted to the presence of such toxic elements by reducing their metabolism and most likely their number as can be hypothesized from the density of the DAPI-labelled nuclei. As a confirmation of the cytotoxicity induced by the generated active species, the specific CellRox fluorescent dye was detected

in the majority of the cells exposed to the H_2O_2-doped medium, thus giving a confirmation of the suitability of the proposed protocol to resemble the raise of a pro-inflammatory event after implantation.

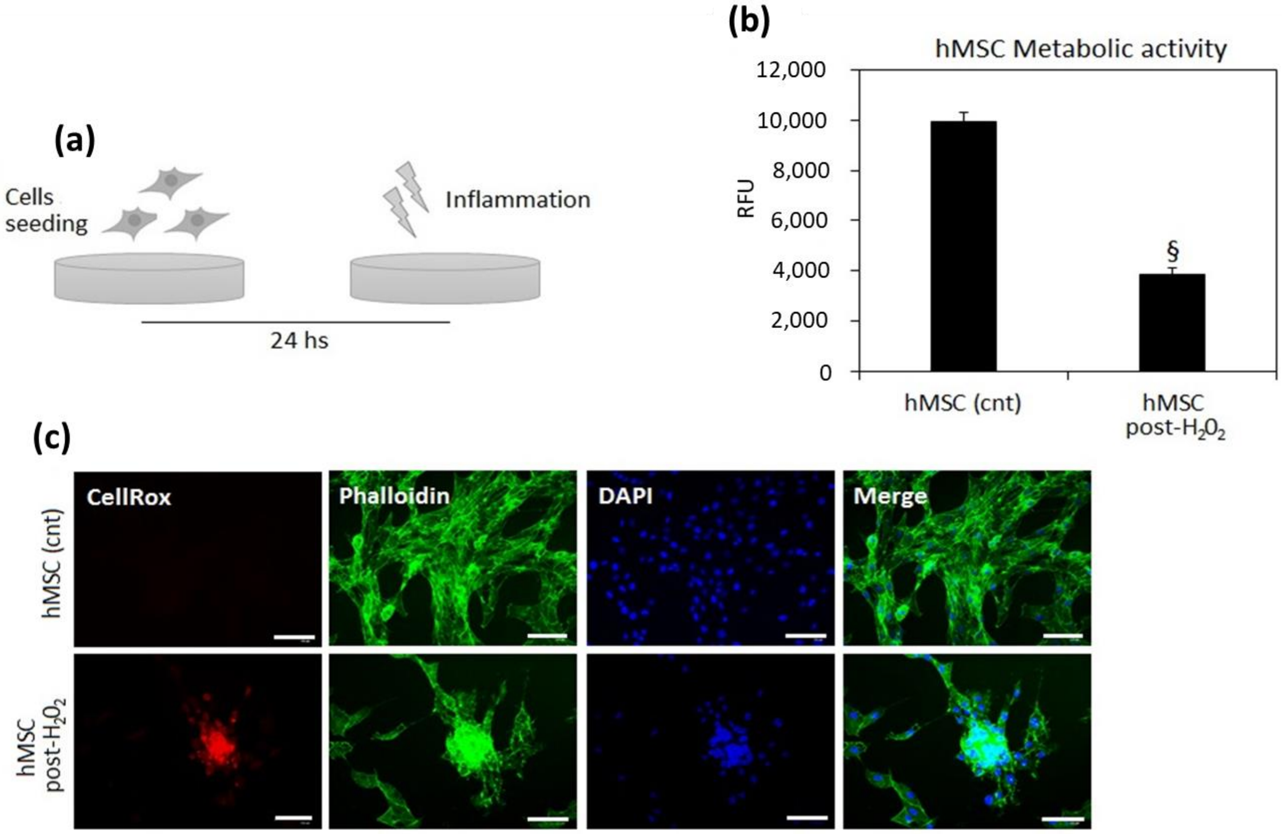

Figure S2. *The use of H_2O_2 to generate toxic active species resembling an inflammatory environment (**a**) determined a significant reduction of the cells' metabolism in comparison to the untreated controls ((**b**), p<0.05 indicated by §). As a confirmation, toxic active species were detected in the majority of the cells exposed to H_2O_2-doped medium ((**c**), positive cells stained in red by CellRox dye). Bars represent means±dev.st, replicates=3. Images bar scale=125 μm.*
